# Prevalence and 11-year incidence of common eye diseases and their relation to health-related quality of life, mental health, and visual impairment

**DOI:** 10.1007/s11136-021-02817-1

**Published:** 2021-03-23

**Authors:** Petri K. M. Purola, Janika E. Nättinen, Matti U. I. Ojamo, Seppo V. P. Koskinen, Harri A. Rissanen, Päivi R. J. Sainio, Hannu M. T. Uusitalo

**Affiliations:** 1 Department of Ophthalmology, Faculty of Medicine and Health Technology, Tampere University, Arvo building, 33014 Tampere, Finland; 2 Finnish Register of Visual Impairment, Finnish Federation of the Visually Impaired, Marjaniementie 74, 00930 Helsinki, Finland; 3 Finnish Institute for Health and Welfare, Mannerheimintie 166, 00270 Helsinki, Finland; 4 Tays Eye Center, Tampere University Hospital, Biokatu 14, 33520 Tampere, Finland

**Keywords:** Eye disease, Health-related quality of life, Incidence, Mental health, Prevalence, Visual impairment

## Abstract

**Purpose:**

To study the prevalence and incidence of the most common eye diseases and their relation to health-related quality of life (HRQoL), depression, psychological distress, and visual impairment in the aging population of Finland.

**Methods:**

Our study was based on two nationwide health surveys conducted in 2000 and 2011. Eye disease status data were obtained from 7379 and 5710 individuals aged 30 + years, of whom 4620 partook in both time points. Both surveys included identical indicators of HRQoL (EuroQol-5 Dimension [EQ-5D], 15D), depression (Beck Depression Inventory [BDI]), psychological distress (General Health Questionnaire-12 [GHQ-12]), visual acuity, and self-reported eye diseases. We assessed the impact of known eye diseases on these factors, adjusted for age, gender, and co-morbidities.

**Results:**

Prevalence of self-reported eye diseases was 3.1/2.7% for glaucoma, 8.1/11.4% for cataract, and 3.4/3.8% for retinal degeneration in 2000 and 2011, and the average incidence between 2000 and 2011 was 22, 109, and 35 /year/10,000 individuals, respectively. These eye diseases were associated with a significant decrease in EQ-5D and 15D index scores in both time points. BDI and GHQ-12 scores were also worsened, with some variation between different eye diseases. Impaired vision was, however, the strongest determinant of declined HRQoL. During the 11-year follow-up the effect of eye diseases on HRQoL and mental health diminished.

**Conclusion:**

Declined HRQoL associated with eye diseases is more related to impaired vision than the awareness of the disease itself, and this declining effect diminished during the follow-up. Therefore, information directed to the public on the risks and prevention of blindness can and should be strengthened to prevent the deleterious effects of visual impairment.

**Supplementary Information:**

The online version contains supplementary material available at 10.1007/s11136-021-02817-1.

## Plain English summary

The prevalence of vision-threatening diseases, such as glaucoma, cataract, and age-related macular degeneration, is likely to increase in developed countries due to aging population and rising life expectancy. Decreased vision is known to worsen the quality of life in eye disease patients. However, a majority of the research on the connection of quality of life with vision and eye diseases has been based on relatively small study populations and vision-specific questionnaires. In this respect, generic instruments could improve the comparability and generalization of the results. In this study, we have evaluated the prevalence and incidence of the most common eye diseases and their impact on generic quality of life in the Finnish adult population during 11 years. This study indicates that even though the prevalence of vision-threatening diseases is increasing, their impact on quality of life is diminishing. The impact of eye diseases on quality of life is related to the impaired vision rather than the awareness of the disease itself. Thus, the information directed to the public about eye diseases and their risks should be strengthened to promote early diagnosis and prevent the declining effect of visual impairment on quality of life and increasing health care costs.

## Introduction

The aging population, rising life expectancy, and unfavorable changes in lifestyle, such as unhealthy eating habits and decreased exercise, in developed countries are likely to increase the prevalence of vision-threatening diseases in the future [1–3]. The most common causes of visual impairment include glaucoma, cataract, and age-related macular degeneration that mainly affect older adults, and inherited retinal diseases affecting young population [4–6], although the permanent deterioration of visual acuity (VA) caused by cataract can usually be prevented with modern surgery [4, 7].

Decreased VA can significantly affect the quality of life (QoL) of an individual even before the individual has become visually impaired (VA ≤ 0.25, Snellen decimal equivalent) [8] and, in fact, the awareness of an eye disease, such as glaucoma, is thought to reduce QoL through the fear of declining vision before the loss of VA affects the patient’s life [7, 9–11]. However, a majority of the previously conducted research on the connection of QoL with VA and eye diseases has been based on relatively small study samples that may not be representative on larger populations [9, 12–14]. Furthermore, many studies have measured QoL using vision-related assessments [15, 16], but more generic instruments could allow better comparison to other diseases and defects. Therefore, we aimed to study the prevalence and incidence of glaucoma, cataract, retinal degenerations (RDs), and their relation to decreased VA and visual impairment using data from two cross-sectional surveys and an 11-year longitudinal follow-up study that are representative of the Finnish adult population. Furthermore, we aimed to study their impact on QoL and mental health using generic instruments included in the surveys that assess health-related quality of life (HRQoL), depression, and psychological distress.

## Materials and methods

### Study population and design

We utilized two nationwide health examination surveys performed in Finland. They were carried out by the Finnish Institute for Health and Welfare, the first one in 2000–2001 and a follow-up in 2011 [17, 18]. In both surveys, the information on eye diseases and co-morbidities was collected in face-to-face interviews, whereas the assessment of HRQoL and mental health was based on self-administered questionnaires. The Health 2000 Survey analyzed a sample of 9922 adults aged 18 years or over living in mainland Finland. The sample was selected by a stratified two-stage cluster sampling design. The Health 2011 Survey analyzed a sample of all living members of the Health 2000 sample who had not refused to be contacted, aged 29 years or over, and a new sample of 1994 young adults aged 18 to 28 years. For this study, we only included participants aged 30 years or over in both cross-sectional and longitudinal samples. Both surveys provided a probability-clustered sampling and weighting scheme that estimates health statistics that are representative of Finnish adult population aged 30 years or over at the time of sampling [19, 20]. In addition, the scheme accounts for the oversampling of people aged 80 years or over in 2000 by doubling the sample fraction. The unweighted participation rate was 93% in the Health 2000 Survey while in the follow-up it was 73%. Separate weights were applied for the surveys to produce results representing the Finnish population at each time points [21].

### Self-reported eye disease status

Both surveys included an interview with the following questions on eye diseases: “Has a doctor diagnosed one of the following diseases: cataract, glaucoma, retinal degeneration, or other visual defect or eye trauma?” Only the individuals who had replied “yes” or “no” to at least one of these questions were chosen for the further analyses, classified as “eye disease status known”. Individuals who had only answered “no” to this set of questions were considered to not have eye diseases. There was also self-reported information on previously performed cataract operations. Only unoperated cataract patients were included in the evaluation of HRQoL, mental health, and VA, as cataract surgeries improve VA and have been demonstrated to improve QoL as well [22].

### Assessment of health-related quality of life

HRQoL scores were evaluated using generic preference-based 3-level version of EuroQol-5 Dimension (EQ-5D-3L, later referred as EQ-5D) and 15D questionnaires that assess physical, psychological, and social functioning and well-being [23, 24]. 15D is a self-administrated measure of HRQoL comprising one question for each of the 15 dimensions—mobility, vision, hearing, breathing, sleeping, eating, speech, excretion, usual activities, mental function, discomfort and symptoms, depression, distress, vitality, and sexual activity. Each question contains five answer options on a scale of 1 (no difficulties) to 5 (extreme difficulties). A single index score is obtained by weighting the obtained scores with population-based preference weights based on an application of the multi-attribute utility theory [25]. In this study, the 15D was weighted using Finnish preference weights with a scale of 0 (representing HRQoL equal to being dead) to 1 (representing the best possible HRQoL). Mean change/difference of ≥ 0.015 was considered to be clinically meaningful [26].

EQ-5D contains one question for each of the five dimensions: mobility, self-care, usual activities, pain/discomfort, and anxiety/depression. Each question contains three answer options on a scale of 1 (no difficulties) to 3 (extreme difficulties), and they can be converted into EQ-5D index scores on a scale identical to the 15D index score. In this study, EQ-5D was weighted using UK time trade-off weights on a scale between − 0.59 (representing HRQoL equal to being dead) and 1 (representing the best possible HRQoL) to improve comparability with other populations [27]. Mean change/difference of ≥ 0.07 was considered to be clinically meaningful [28].

### Assessment of mental health

The state of mental health was assessed using two self-report questionnaires, Beck Depression Inventory (BDI) and General Health Questionnaire-12 (GHQ-12). In the first survey, BDI-21 comprising 21 questions was used to evaluate depression, whereas in the follow-up survey a shorter version, BDI-13, containing 13 questions, was used [29, 30]. A total score was calculated for both questionnaires with a scale of 0–63 for BDI-21 and 0–39 for BDI-13, where higher points indicate major depression. Total scores of ≥ 10 for BDI-21 and ≥ 5 for BDI-13 were used as cut-off points to categorize an individual as having depression [31].

GHQ-12 is a questionnaire comprising 12 questions that evaluate different dimensions of psychological distress, including depression, anxiety, social interaction, and confidence [32, 33]. The answers were dichotomized according to whether difficulties were presented or not (0 = no, 1 = yes). A total score with a scale of 0–12 was calculated using the dichotomized points, with 12 representing the highest psychological distress. A total score of > 3 was considered as indicative of psychological distress [17, 18].

### Visual acuity tests

In both surveys, the distance VA was measured by a study nurse binocularly at 4 m with current visual correction. Illumination was set to ≥ 350 lx on the modified logMAR letter chart published by Precision Vision [19, 20, 34]. All VA values are presented as Snellen decimal equivalents. Low VA values outside the modified logMAR letter chart that could not be determined were reported as 0.01. The classified VA values were following: VA ≥ 1.0 (good vision), VA 0.63–0.8 (adequate vision), VA 0.32–0.5 (weak vision), VA 0.125–0.25 (impaired vision), and VA < 0.1 (severe vision loss or blindness) [8]. Habitual binocular distance VA ≤ 0.25 was considered as impaired vision. We found the binocular evaluation of VA important because the relation of vision and HRQoL was studied.

### Co-morbidities

Common diseases assessed in the interview (data available from 7371 to 7385 and 5714 to 5720 participants in 2000 and 2011) were accounted for their potential impact on the HRQoL and mental health. The diseases were classified into major co-morbidity groups according to Taipale and colleagues [8]: heart diseases (myocardial infarction, angina pectoris, heart failure, arrhythmias, and “other heart disorders”); respiratory diseases (asthma, chronic obstructive pulmonary disease, chronic bronchitis, and “other pulmonary disease”); vascular diseases (stroke and varicose veins in lower limbs); musculoskeletal conditions (rheumatoid arthritis, osteoarthrosis, fractures, and osteoporosis); psychiatric conditions (psychotic disorders, depression, anxiety, psychoactive substance abuse, and “other psychiatric disease”). Furthermore, hypertension, diabetes, Parkinson’s disease, and unspecified cancer were included as separate groups.

Co-morbidity status was determined according to Taipale and colleagues [8] so that individuals were considered to have co-morbidity if they reported any of the conditions included in the co-morbidity group. When analyzing new incident diagnoses during the follow-up period, each condition was scrutinized in 2000 baseline and in 2011 follow-up. If the subject reported at least one new condition included in the co-morbidity group in 2011, they were classified as having incident co-morbidity, regardless of the presence of other conditions of that specific co-morbidity group at baseline.

### Statistical analyses

The data were analyzed using R software version 3.5.1 [35], and it included both cross-sectional survey samples for all cross-sectional and longitudinal analyses. The sampling design, the oversampling of individuals aged 80 years or over, and the loss to follow-up were accounted for by using Survey package 3.37 for R [36] and weighting scheme calculated by the Finnish Institute for Health and Welfare.

For the prevalence and incidence analyses, population totals and ratios were estimated using functions *svytotal* and *svyratio* included in the Survey package. Individuals with missing data in analyzed variables were excluded. As the distribution of the continuous variable data was skewed, Mann–Whitney *U* test was used for the between-group comparisons. The impact of age, gender, eye diseases, impaired distance VA, and co-morbidities on HRQoL and mental health were estimated through linear regression, and standardized regression coefficients were calculated using lm.beta package 1.5-1 for R [37]. Multicollinearity in regression analyses was measured through variance inflation factors using car package 2.1-5 for R [38, 39]. Odds ratios (ORs) with 95% confidence intervals were calculated using logistic regression analysis, adjusted for age, gender, and co-morbidities. For all analyses, a two-tailed *p* value of < 0.05 was considered to be statistically significant.

## Results

### Eye disease status of the participants

**Fig. 1:**
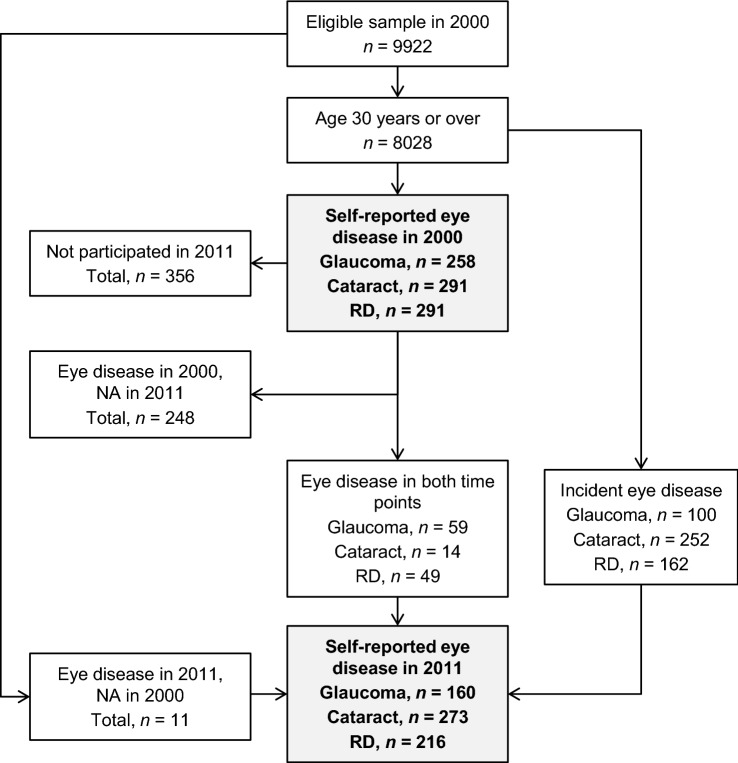
Flow chart of glaucoma, unoperated cataract, and retinal degeneration (RD) status. *NA* not applicable

Figure 1 presents the number of the individuals with self-reported eye disease in the two surveys that were included in the analyses. In total, 8028 individuals aged 30 years or over had been invited in the 2000 survey and 8006 in the 2011 survey. Eye disease status data was obtained from 7379 and 5710 individuals, of whom 4620 took part in both time points and were included in the 11-year follow up. Table 1 shows the number, mean age, and the gender distribution of the survey samples, individuals with/without eye diseases, and individuals with impaired/good distance VA in both surveys and in the 2011 follow-up. It also includes the number of individuals with eye disease status known who had available data on HRQoL, mental health, and distance VA. The data in all analyses were compared between individuals with eye diseases and those with no eye diseases, and individuals with impaired distance VA and those with good distance VA.

**Table 1:** Summary of the participants aged 30 years or over in Health 2000 and 2011 studies

	2000			2011			11-year follow-up group in 2011		
	*n*	Mean age (SD)	% women	*n*	Mean age (SD)	% women	*n*	Mean age (SD)	% women
Eligible sample	8028	54.2 (16.2)	54.7	8006	55.3 (15.6)	53.0	6360	60.6 (12.9)	55.5
Eye disease status known	7379	54.2 (16.1)	55.2	5710	55.6 (14.6)	55.4	4620	60.0 (12.1)	55.6
No eye diseases	4793	52.1 (15.6)	51.8	4067	53.3 (14.2)	53.2	3122	58.3 (11.7)	53.2
Glaucoma	258	71.1 (13.6)	75.2	160	72.0 (11.2)	66.9	159	72.2 (10.8)	66.7
Cataract, all	740	76.7 (10.4)	73.6	663	73.8 (10.1)	63.7	654	74.1 (9.6)	63.6
Cataract, unoperated	291	74.3 (10.1)	74.9	273	71.1 (9.0)	64.8	268	71.4 (8.5)	64.9
Cataract, operated	449	78.2 (10.3)	72.8	390	75.7 (10.5)	62.3	386	76.0 (9.9)	62.5
RD	291	73.5 (12.4)	67.7	216	73.1 (12.0)	62.0	211	73.7 (11.0)	62.0
Distance VA measured	6644	53.6 (15.5)	55.3	4554	56.5 (14.1)	55.7	3804	60.1 (11.9)	55.5
Good distance VA (≥ 1.0)	4943	48.6 (12.2)	53.6	3678	53.5 (12.7)	55.7	3002	57.4 (10.3)	54.9
Impaired distance VA (≤ 0.25)	147	80.0 (11.7)	74.1	52	76.8 (13.7)	61.5	45	77.9 (13.1)	62.2
EQ-5D index score available	6131	53.5 (15.7)	55.9	4024	55.8 (13.9)	56.3	3082	59.4 (11.7)	56.8
15D index score available	6149	53.2 (15.2)	55.7	4212	56.3 (13.8)	56.2	3460	59.8 (11.6)	56.1
BDI total score available	6297	52.7 (14.9)	55.0	4300	56.1 (13.8)	56.0	3562	59.6 (11.5)	55.7
GHQ-12 total score available	6530	53.2 (15.3)	55.1	4445	56.2 (14.0)	55.8	3685	59.8 (11.7)	55.7

The 11-year follow-up group includes the individuals who participated in both years (aged 30 years or over) and the eye disease status of these individuals in 2011

*RD* retinal degeneration, *SD* standard deviation, *VA* visual acuity

### Prevalence and incidence of eye diseases

**Fig. 2:**
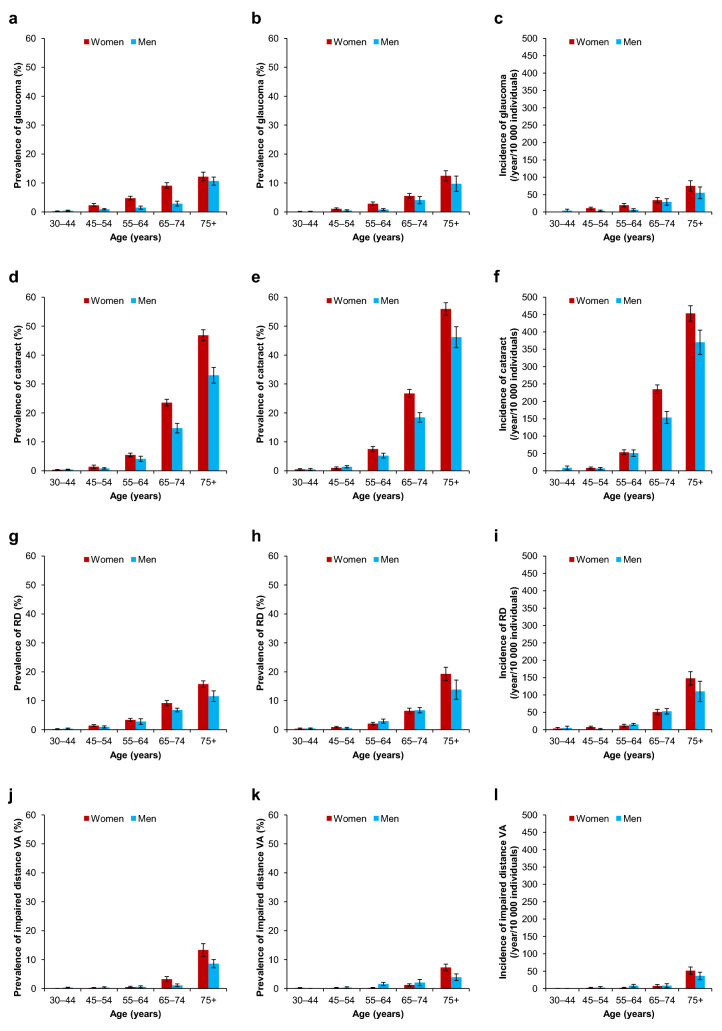
Prevalence of glaucoma, cataract, retinal degeneration (RD), and impaired distance visual acuity (VA; ≤ 0.25) in 2000 and 2011, and their incidence 2000–2011 (with 95% confidence intervals) in the Finnish population aged 30 years or over by gender and age. Prevalence of glaucoma in 2000 (**a**) and 2011 (**b**), and incidence 2000–2011 (**c**); prevalence of cataract in 2000 (**d**) and 2011 (**e**), and incidence 2000–2011 (**f**); prevalence of RD in 2000 (**g**) and 2011 (**h**), and incidence 2000–2011 (**i**); prevalence of visual impairment in 2000 (**j**) and 2011 (**k**), and incidence 2000–2011 (**l**)

The estimated prevalence and incidence of glaucoma, cataract, RD, and visual impairment in the Finnish adult population is shown in Table 2. The prevalence of cataract and RD increased between the time points, whereas glaucoma and visual impairment decreased. The prevalence and incidence of all eye diseases and visual impairment increased with age, and they appeared to be more common in women, particularly in age group 75 + years (Fig. 2).

**Table 2:** Estimated prevalence and incidence with 95% confidence intervals (CIs) of eye diseases and visual impairment in the Finnish population aged 30 years or over in 2000 and 2011

	2000		2011		Incidence 2000–2011	
	*N *(95% CI)	Prevalence % (95% CI)	*N* (95% CI)	Prevalence % (95% CI)	*N* (95% CI)	*N*/year/10,000 individuals (95% CI)
Glaucoma	100,517 (76,226–124,808)	3.10 (2.95–3.26)	83,453 (64,288–102,618)	2.70 (2.47–2.93)	52,026 (40,359–63,693)	22 (20–23)
Cataract, all	262,927 (200,002–325,852)	8.11 (7.76–8.48)	353,082 (270,532–435,632)	11.41 (10.88–11.94)	257,658 (196,158–319,158)	109 (104–114)
Cataract, unoperated	107,955 (79,476–136,434)	3.50 (3.23–3.77)	140,120 (108,073–172,167)	4.86 (4.60–5.12)	122,239 (93,419–151,059)	55 (52–59)
RD	111,652 (87,115–136,189)	3.45 (3.29–3.61)	118,285 (88,207–148,363)	3.83 (3.46–4.20)	83,843 (61,808–105,878)	35 (31–38)
Impaired distance VA (≤ 0.25)	48,405 (34,479–62,331)	1.58 (1.40–1.76)	31,275 (23,799–38,751)	1.27 (1.13–1.41)	21,134 (15,506–26,762)	10 (8–12)

*RD* retinal degeneration, *VA* visual acuity

### Cross-sectional impact of eye diseases on health-related quality of life, mental health, and visual acuity

**Fig. 3:**
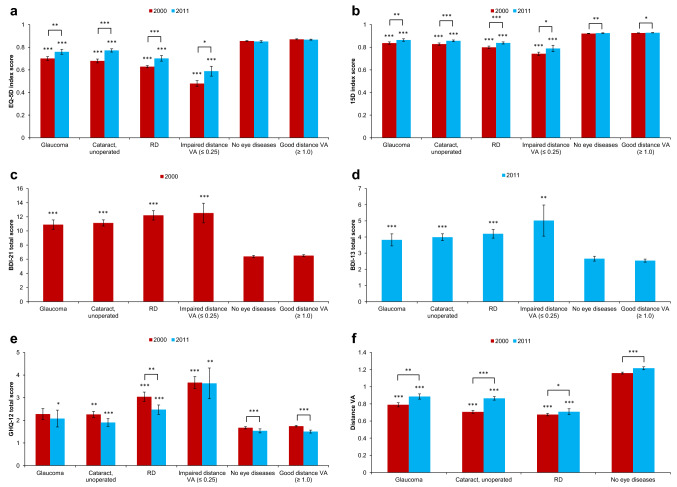
Mean values (with standard error bars) of health-related quality of life index scores (**a**, **b**), mental health total scores (**c**–**e**), and distance visual acuity (VA; **f**) in both time points. When calculating statistical significance (Mann–Whitney U test), eye disease groups were tested against individuals with no eye diseases, and individuals with impaired distance VA were tested against those with good distance VA within the same year. In addition, mean values were compared between time points in each group. *RD* retinal degeneration. *Denotes statistical significance with *p* < 0.05. **Denotes statistical significance with *p* < 0.01. ***Denotes statistical significance with *p* < 0.0001

EQ-5D and 15D index scores were lower (*p* < 0.0001) in individuals with eye disease or visual impairment compared to those with no eye disease or with good distance VA in both time points, indicating lower HRQoL in eye disease patients and visually impaired (Fig. 3). This difference was also clinically meaningful in both time points. However, the mean values of these scores were higher in 2011 than in 2000 in all eye disease groups (*p* < 0.01) and individuals with visual impairment (*p* < 0.05). Individuals with no eye diseases and those with good distance VA had better HRQoL in 2011 only according to 15D score (*p* = 0.0001 and *p* = 0.036, respectively). Moreover, the improvement of mean HRQoL seen in all eye disease groups and those with visual impairment was clinically meaningful between the time points, except for glaucoma with EQ-5D.

All eye disease groups and visually impaired had worse (*p* < 0.0001) BDI-21 total scores compared to individuals with no eye diseases or those with good distance VA (Fig. 3). Similar difference was found for BDI-13 total scores in all eye disease groups (*p* < 0.0001) and visually impaired (*p* = 0.002). Because the scales of the BDI questionnaires between the time points were not comparable, the mean change between time points was not evaluated. The effect of various eye diseases on GHQ-12 varied: it was most severe in RD (*p* < 0.0001 in both time points) and least severe in glaucoma (*p* = 0.037, only in 2011). Only individuals with RD (*p* = 0.004) and those with no eye disease or with good distance VA (*p* < 0.0001) showed better GHQ-12 scores in 2011 than in 2000. All eye disease groups had worse (*p* < 0.0001) distance VA compared to those with no eye diseases in both time points. All groups showed better mean distance VA in 2011 than in 2000. Overall, RD was associated with lowest scores in all these parameters compared to other eye diseases in 2000, but in 2011 this difference was significant only in EQ-5D (*p* = 0.010) and distance VA (*p* < 0.0001). However, visual impairment showed the highest impact on these parameters compared to all eye diseases in both time points, excluding BDI-21 and BDI-13, in which no significant difference was found between eye diseases and visual impairment.

### Cross-sectional impact of eye diseases on the individual dimensions of health-related quality of life

The individual HRQoL dimensions were evaluated using ORs by comparing those with difficulties to those without difficulties. In addition, mental health was assessed by using the cut-off points for GHQ-12 and BDI total scores. For HRQoL, the most affected dimensions in individuals with eye disease and those with visual impairment were usual activities and mobility in EQ-5D, and vision, usual activities, and vitality in 15D (Table 3). There was variation in how the dimensions were affected between the eye diseases: among those with RD the majority of the individual dimensions were affected in both EQ-5D and 15D, whereas among those with unoperated cataract none of the EQ-5D dimensions differed from those with no eye diseases, even though the index score was significantly lower. Pain/discomfort and anxiety/depression in EQ-5D was only affected in those with RD in 2000, and anxiety/depression in EQ-5D was affected in visually impaired in both time points. The vision dimension in 15D was most affected in all eye diseases and visually impaired. Individuals with visual impairment showed high odds for having difficulties, including eight-fold increase in usual activities (EQ-5D) and sixfold increase in self-care (EQ-5D) in 2000 compared to individuals with good distance VA. Self-care (EQ-5D) was affected in individuals with visual impairment but not in those with eye disease. For mental health, depression in 2000 (BDI-21 ≥ 10) was more prevalent among individuals with RD and unoperated cataract compared to those with no eye diseases. In 2011, BDI-13 was not significantly affected in any of the groups. Psychological distress (GHQ-12 > 3) was found more prevalent among individuals with RD in 2000 and visual impairment in both time points.

**Table 3:** Adjusted odds ratios (ORs) of EQ-5D dimensions, five most affected 15D dimensions, and BDI and GHQ-12 total scores indicative of depression or psychological distress compared to those with no eye diseases or with good distance visual acuity (VA)

	EQ-5D dimensions (95% CI)					Five most affected 15D dimensions (95% CI)					Mental health (95% CI)	
	Mobility	Self-care	Usual activities	Pain/discomfort	Anxiety/ depression	Vision	Usual activities	Mobility	Vitality	Depression	BDI-21 ≥ 10 in 2000, BDI-13 ≥ 5 in 2011	GHQ− 12 > 3
2000												
Glaucoma	1.44 (0.92–2.28)	0.99 (0.71–1.40)	1.61* (1.16–2.24)	1.17 (0.95–1.45)	1.19 (0.71–2.02)	2.25** (1.64–3.09)	1.71* (1.16–2.51)	1.61 (1.00–2.58)	1.20 (0.80–1.78)	1.37 (1.01–1.86)	1.40 (1.03–1.91)	1.08 (0.78–1.50)
Cataract, unoperated	1.11 (0.78–1.59)	0.76 (0.59–0.96)	1.11 (0.84–1.48)	1.36 (0.94–1.96)	1.06 (0.70–1.61)	2.38** (1.57–3.60)	1.19 (0.81–1.76)	1.25 (0.89–1.75)	1.46* (1.14–1.88)	0.98 (0.69–1.41)	1.60* (1.15–2.24)	0.88 (0.73–1.07)
RD	2.23** (1.60–3.09)	1.13 (0.87–1.45)	1.81** (1.38–2.39)	2.51** (1.65–3.83)	1.62* (1.12–2.34)	4.45*** (3.35–5.91)	2.45** (1.67–3.57)	1.83** (1.30–2.56)	2.23** (1.55–3.20)	1.70* (1.25–2.30)	2.00** (1.56–2.57)	1.63** (1.26–2.11)
Impaired distance VA (≤ 0.25)	2.70** (1.60–4.55)	6.54** (4.05–10.6)	8.44** (4.90–14.5)	1.11 (0.66–1.90)	3.72** (1.85–7.48)	22.21*** (14.6–33.8)	2.65* (1.37–5.14)	2.77** (1.99–3.86)	2.51** (1.51–4.17)	1.84 (1.06–3.20)	1.49 (0.83–2.71)	2.90** (2.18–3.86)
2011												
Glaucoma	1.53* (1.12–2.07)	1.19 (0.74–1.89)	1.11 (0.60–2.05)	1.07 (0.68–1.68)	0.77 (0.41–1.42)	1.86 (0.94–3.68)	1.84* (1.17–2.89)	1.30 (1.00–1.69)	1.62* (1.09–2.42)	1.14 (0.88–1.45)	1.35 (0.86–2.11)	1.24 (0.69–2.23)
Cataract, unoperated	1.25 (0.91–1.71)	0.56 (0.34–0.94)	0.75 (0.52–1.10)	0.92 (0.61–1.39)	0.58 (0.31–1.09)	2.30** (1.54–3.43)	1.53* (1.12–2.10)	1.51* (1.07–2.12)	0.95 (0.67–1.37)	1.47 (0.98–2.19)	1.23 (0.90–1.69)	0.90 (0.57–1.43)
RD	1.19 (0.89–1.60)	1.22 (0.73–2.04)	1.39 (0.96–2.01)	1.33 (0.89–1.98)	0.96 (0.57–1.61)	3.55*** (2.71–4.66)	1.89* (1.31–2.71)	1.11 (0.69–1.77)	1.48* (1.13–1.94)	1.09 (0.79–1.49)	1.29 (0.77–2.16)	1.49 (1.04–2.12)
Impaired distance VA (≤ 0.25)	2.24* (1.15–4.37)	6.13** (3.23–11.6)	4.64** (2.18–9.88)	0.90 (0.44–1.81)	2.91* (1.44–5.88)	44.51** (16.9–117.1)	3.28* (1.53–7.04)	2.04 (0.86–4.84)	2.31 (0.97–5.52)	1.38 (0.78–2.43)	1.65 (0.87–2.51)	3.23** (1.88–5.54)

The ORs and 95% confidence intervals (CIs) were estimated through logistic regression analysis adjusted for age, gender, and co-morbidities. Bolded values denote statistically significant (*p* < 0.05) ORs compared to reference group. Reference group (OR = 1.0) for individuals with eye diseases included those with no eye diseases, and reference group for individuals with impaired distance VA included those with good distance vision (VA ≥ 1.0). *RD* retinal degeneration

*Denotes statistical significance with *p* < 0.05

**Denotes statistical significance with *p* < 0.01

***Denotes statistical significance with *p* < 0.0001

### Cross-sectional analyses corrected with age, gender, and co-morbidities

The effect of the awareness of the eye diseases on the HRQoL and mental health was evaluated using linear regression analyses, including age, gender, and co-morbidities (Tables 4 and 5). After these corrections, the impact of impaired distance VA on HRQoL was more significant than any of the eye diseases. Only RD showed significant impact on 15D, GHQ-12, and BDI-21 of all eye diseases in 2000, whereas in 2011 only unoperated cataract showed significant impact on 15D of all eye diseases. In addition to visual impairment, psychiatric disorder and Parkinson’s disease had high impact on HRQoL. However, the overall effect and/or association of all these diseases and visual impairment on HRQoL and mental health were lower in 2011 than in 2000. No significant change was observed in the outcome when only statistically significant (*p* < 0.05) factors were included as explanatory variables in stepwise-insertion analysis. Multicollinearity ranged from 1.007 to 1.520 denoting no or very little multicollinearity.

**Table 4:** Multivariable linear regression analysis examining the impact of eye diseases, visual impairment, age, gender, and co-morbidities on EQ-5D and 15D index values, and GHQ-12 and BDI-21 total scores in 2000

	Change in EQ-5D (*n* = 5643)		Change in 15D (*n* = 5777)		Change in GHQ-12 (*n* = 6064)		Change in BDI-21 (*n* = 5886)	
	B coefficients	Beta coefficients	B coefficients	Beta coefficients	B coefficients	Beta coefficients	B coefficients	Beta coefficients
Constant	1.062***		1.035***		1.605*		2.437**	
Age	− 0.003***	− 0.213***	− 0.002***	− 0.259***	− 0.008	− 0.039	0.058**	0.115**
Male gender	0.012**	0.031**	− 0.0004	− 0.002	− 0.148	− 0.025	− 1.23***	− 0.087***
Glaucoma	− 0.007	− 0.005	− 0.008	− 0.013	− 0.021	− 0.001	0.428	0.009
Cataract, unoperated	− 0.013	− 0.012	− **0.017**	− 0.034	− 0.135	− 0.009	0.844	0.022
RD	− 0.047*	− 0.038*	− 0.033*	− 0.057*	0.654*	0.036*	1.713*	0.038*
Impaired distance VA (≤ 0.25)	− 0.210***	− 0.125***	− 0.083**	− 0.099**	1.464*	0.055*	1.091	0.016
Heart disease	− 0.041**	− 0.069**	− 0.032***	− 0.112***	0.256*	0.029*	0.804*	0.037*
Pulmonary disease	− 0.022*	− 0.044*	− 0.024**	− 0.103**	0.305*	0.042*	1.081**	0.062**
Vascular disease	− 0.025*	− 0.047*	− 0.007	− 0.028	0.269*	0.035*	0.476	0.025
Musculoskeletal condition	− 0.059***	− 0.148***	− 0.017**	− 0.093**	0.361*	0.062*	1.167**	0.083**
Hypertension	− 0.011*	− 0.024*	− 0.007*	− 0.036*	0.145	0.023	0.488	0.032
Diabetes	− 0.073**	− 0.081**	− 0.033**	− 0.077**	0.327	0.025	1.577*	0.049*
Psychiatric disorder	− 0.129***	− 0.219***	− 0.068***	− 0.247***	2.118***	0.246***	6.635***	0.319***
Parkinson’s disease	− 0.195*	− 0.059*	− 0.072**	− 0.041**	2.153*	0.044*	3.194	0.026
Cancer	− 0.013	− 0.013	− **0.018**	− 0.042	0.352	0.025	1.240	0.037
*R*^*2*^	0.283***	0.283***	0.359***	0.359***	0.088***	0.088***	0.200***	0.200***
Adjusted *R*^*2*^	0.281***	0.281***	0.358***	0.358***	0.086***	0.086***	0.198***	0.198***

The unstandardized B coefficients show the magnitude of the impact on health-related quality of life and mental health, while the standardized Beta coefficients allow the comparison of the explanatory variables with each other. Clinically meaningful B coefficients are bolded (≥ 0.07 for EQ-5D and ≥ 0.015 for 15D). *RD* retinal degeneration, *VA* visual acuity

*Denotes statistical significance with *p* < 0.05

**Denotes statistical significance with *p* < 0.01

***Denotes statistical significance with *p* < 0.0001

**Table 5:** Multivariable linear regression analysis examining the impact of eye diseases, visual impairment, age, gender, and co-morbidities on EQ-5D and 15D index values, and GHQ-12 and BDI-13 total scores in 2011

	Change in EQ-5D (*n* = 3763)		Change in 15D (*n* = 3936)		Change in GHQ-12 (*n* = 4148)		Change in BDI-13 (*n* = 4018)	
	B coefficients	Beta coefficients	B coefficients	Beta coefficients	B coefficients	Beta coefficients	B coefficients	Beta coefficients
Constant	1.009***		1.002***		1.932***		1.356*	
Age	− 0.001**	− 0.112**	− 0.0008**	− 0.134**	− 0.020**	− 0.098**	0.007	0.022
Male gender	0.009	0.025	0.0007	0.004	− 0.155	− 0.029	− 0.303*	− 0.039*
Glaucoma	− 0.024	− 0.019	− 0.005	− 0.010	0.377	0.020	0.648	0.024
Cataract, unoperated	0.002	0.003	− 0.023**	− 0.065**	0.043	0.004	0.410	0.024
RD	− 0.004	− 0.004	− 0.009	− 0.017	0.081	0.005	− 0.426	− 0.017
Impaired distance VA (≤ 0.25)	− 0.126*	− 0.058*	− 0.091*	− 0.080*	1.066	0.032	2.307	0.044
Heart disease	− 0.042**	− 0.082**	− 0.027***	− 0.115***	0.676**	0.087**	1.056**	0.094**
Pulmonary disease	− 0.040**	− 0.078**	− 0.026**	− 0.112**	0.267	0.035	0.310	0.028
Vascular disease	− 0.012	− 0.022	− 0.004	− 0.017	0.129	0.017	− 0.083	− 0.007
Musculoskeletal condition	− 0.068***	− 0.194***	− 0.018**	− 0.112**	0.373*	0.070*	0.449*	0.058*
Hypertension	− 0.039**	− 0.100**	− 0.016**	− 0.090**	0.321*	0.055*	0.362*	0.043*
Diabetes	− 0.040	− 0.060	− 0.026*	− 0.086*	0.481	0.048	0.344	0.023
Psychiatric disorder	− 0.127***	− 0.226***	− 0.074***	− 0.286***	2.425***	0.285***	4.992***	0.406***
Parkinson’s disease	− **0.216**	− 0.069	− 0.106*	− 0.066*	1.473	0.034	3.209	0.045
Cancer	0.006	0.008	− 0.005	− 0.015	0.097	0.009	− 0.050	− 0.003
*R*^*2*^	0.187***	0.187***	0.250***	0.250***	0.114***	0.114***	0.188***	0.188***
Adjusted *R*^*2*^	0.183***	0.183***	0.247***	0.247***	0.111***	0.111***	0.185***	0.185***

The unstandardized B coefficients show the magnitude of the impact on health-related quality of life and mental health, while the standardized Beta coefficients allow the comparison of the explanatory variables with each other. Clinically meaningful B coefficients are bolded (≥ 0.07 for EQ-5D and ≥ 0.015 for 15D). *RD* retinal degeneration, *VA* visual acuity

*Denotes statistical significance with *p* < 0.05

**Denotes statistical significance with *p* < 0.01

***Denotes statistical significance with *p* < 0.0001

### Longitudinal impact of eye diseases on health-related quality of life and mental health

The longitudinal effect of newly-diagnosed eye diseases on the changes in EQ-5D, 15D, and GHQ-12 during the follow-up was evaluated using linear regression, which also included age, gender, incident co-morbidities, and baseline scores (see table in Online Resource 1). BDI was not included as the different scales of the questionnaires were not fully comparable between the surveys. Incident visual impairment and Parkinson’s disease were not included as their number was low (*n* < 50).

Newly-diagnosed eye diseases had no direct independent association in the change of the dependent variables, except for unoperated cataract which was associated with a small decrease in 15D index score. The highest impact on EQ-5D, 15D, and GHQ-12 change both clinically and statistically was observed in newly diagnosed psychiatric disorder and baseline index/total score. No significant change was observed in the outcome when only statistically significant (*p* < 0.05) factors were included as explanatory variables in stepwise-insertion analysis. Multicollinearity ranged from 1.007 to 1.208, denoting no or very little multicollinearity.

**Fig. 4:**
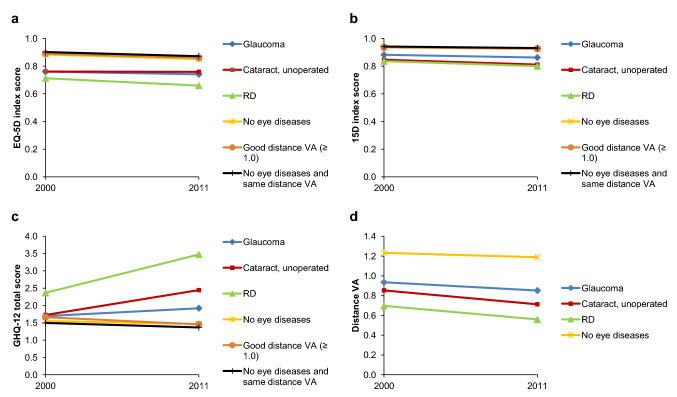
Change in health-related quality of life (**a**, **b**), psychological distress (**c**), and distance visual acuity (VA; d) in individuals with same eye status in both time points. Low scores for EQ-5D and 15D indicate worse quality of life and high score for GHQ-12 worse mental health. For reference, a group of individuals with no eye diseases and same distance VA in both time points was included. *RD* retinal degeneration

**Fig. 5:**
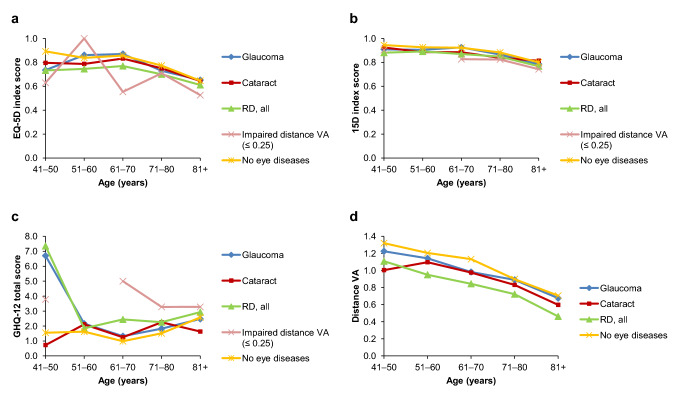
Relation of age to health-related quality of life (**a**, **b**), psychological distress (**c**), and distance visual acuity (VA; **d**) in individuals who had participated in both time points by age and 2011 eye status. Low scores for EQ-5D and 15D indicate worse quality of life and high score for GHQ-12 worse mental health. Few data points for young individuals with impaired VA are missing as the number of these individuals was low. *RD* retinal degeneration

Furthermore, the longitudinal setting was utilized when observing the change in the HRQoL, GHQ-12 scores, and distance VA in individuals who had same eye status in both time points (Fig. 4). Individuals with visual impairment in both time points were not included as their number was low (*n* = 8). All groups, including those with no eye diseases and with good distance VA, showed a small decline in the HRQoL values, with clinically meaningful decline in 15D values in all eye disease groups. All eye disease groups showed worsening in the GHQ-12 total score and all groups showed decrease in the distance VA. The impact of aging was visualized (Fig. 5), which shows that the decline in HRQoL and distance VA, and worsening in GHQ-12 is associated with aging.

### Association between health-related quality of life, mental health, and visual acuity

**Fig. 6:**
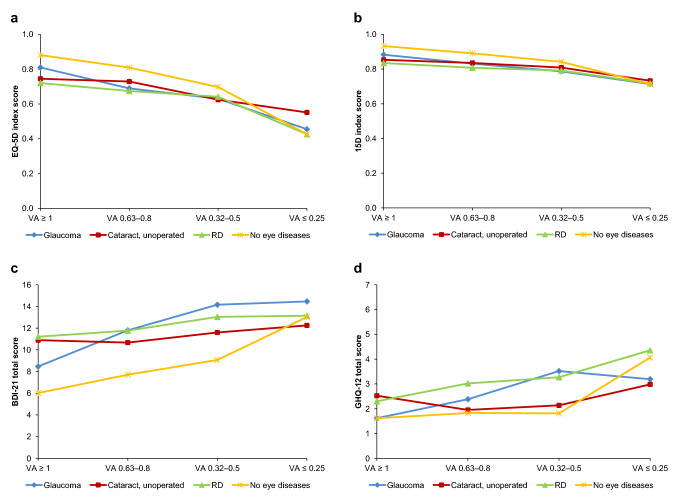
Mean values of health-related quality of life (**a**, **b**) and mental health (**c**, **d**) compared to distance visual acuity (VA) in 2000. *RD* retinal degeneration

**Fig. 7:**
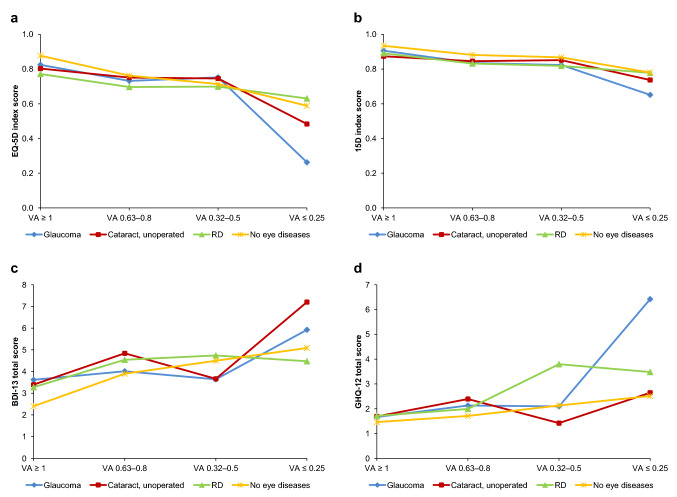
Mean values of health-related quality of life (**a**, **b**) and mental health (**c**, **d**) compared to distance visual acuity (VA) in 2011. *RD* retinal degeneration

Figures 6 and 7 show the overall shape of association between HRQoL, mental health, and distance VA in both time points. The decrease in the HRQoL and the worsening in mental health are associated with decreasing distance VA in all groups, including those with no known eye diseases.

## Discussion

Individuals with eye diseases and visual impairment have lower HRQoL, VA, and worsened mental health compared to individuals without eye diseases and those with good vision. Of all the individual dimensions of the used generic HRQoL instruments, vision, usual activities, vitality, and mobility were most affected. Previous publications have shown that visually impaired people express declined generic HRQoL and vision-related QoL and have more difficulties in the activities of daily living [40–43]. Because vision was significantly affected in all eye diseases and the difficulties in usual activities, vitality, and mobility were prevalent in individuals with visual impairment, the difficulties in these dimensions may be associated with the decreased VA. The worsened mental health in eye diseases may also be associated with the declined VA, as Taipale and colleagues previously showed with identical data set that BDI and VA seem to have a linear connection [8]. Furthermore, increased depression and anxiety have been previously associated with visual impairment, particularly among older adults [42–44]. Li and colleagues reported an association between age-related eye diseases, visual impairment, and declined generic HRQoL similar to our results, although they did not find association with psychological distress [45]. However, they only included individuals aged 65 years or over, and therefore, the results may not be comparable.

The average HRQoL improved between the cross-sectional studies in all eye disease groups and visually impaired individuals, including a clinically meaningful increase between the time points. Individuals without known eye diseases or with good vision showed minor, although clinically non-meaningful, improvement in HRQoL only according to 15D. When evaluating mental health, only those with RD, as well as individuals without eye diseases or with good vision showed improvement between time points according to GHQ-12. These results suggest that the effect of the eye diseases and visual impairment on these factors had decreased between the time points and that the well-being of eye disease patients and visually impaired individuals has increased in 11 years. Similar improvement in the overall well-being in Finland between 2000 and 2011 has been reported previously [18]. This better well-being of patients suffering from eye disease or visual impairment may be due to better availability of health services, aids, and treatment.

When the cross-sectional analyses were corrected with age, gender, and co-morbidities, RD was associated with a small decline in HRQoL and mental health in 2000, and unoperated cataract with HRQoL in 2011 only according to 15D. However, visual impairment showed more significantly declining effect on HRQoL in both time points, indicating that the impaired vision may have a stronger impact on HRQoL than the awareness of the eye disease itself. Similar results were reported by Knudtson et al. [46], who found that decreased visual function appeared to have a significant effect on the decline in QoL irrespective of pathologic reasons, such as age-related eye diseases. In our study, this association of the eye diseases and visual impairment on HRQoL and mental health was lower in 2011 than in 2000. In longitudinal setting, newly diagnosed eye diseases did not appear to have a direct effect on HRQoL or mental health. Similar to present study, Nutheti et al. reported that the effect of cataract and retinal diseases on generic HRQoL was associated with VA, whereas the effect of glaucoma and corneal diseases were independent of VA [9]. This difference in glaucoma could be explained by the many differences in these two populations regarding age, health, and social care systems.

In the longitudinal setting, individuals with or without eye diseases in both time points showed small decline in their HRQoL in contrast to the improvement found in the cross-sectional setting. This decline was most probably related to the fact that the subjects were 11 years older at the end of follow up. Furthermore, all eye disease groups, visually impaired, and individuals with no eye diseases showed negative association between HRQoL, impaired distance VA, and age. Visual impairment has been previously associated with aging [47], and in our study, the prevalence and incidence of impaired vision as well as vision-affecting eye diseases increased with age.

The strengths of this study include a large study sample representing Finnish adult population aged 30 years or over in two cross-sectional surveys and a longitudinal study with a relatively long follow-up of 11 years. As the study population and design were widely collected and comprehensive, the impact of confounding factors was low. Furthermore, our data did not consist of specific patient groups collected from health-care units, which allows better generalization of the results. High proportion of the individuals participated in both surveys, and the overall adherence to present study can be considered to be good, as mentioned previously by Taipale et al. who used identical data set [8]. In addition, loss to follow-up was compensated by applying calibrated weighting scheme [18]. As a valid assessment of HRQoL requires reports directly from patients rather than physicians or other parties, we used generic HRQoL questionnaires in both time points. We did not use vision-related QoL instruments for better comparability and generalization of the results.

There are also potential limitations in our study. First, self-reported instruments, EQ-5D in particular, assess a limited number of dimensions and can be influenced by the subjective nature of QoL [14]. Furthermore, all eye diseases were self-reported, physician-made diagnoses, but the diagnoses were not confirmed by physicians in the study. We were also unable to include visual impairment caused by diminishing visual field, as well as the examination of contrast sensitivity. The number of visually impaired in the longitudinal analyses were rather low. The variation in the age of the participants was large, but we corrected this by adjusting the age in the analyses. The questionnaire did not include data whether cataract patients had uni- or bilateral cataract. However, in most cases, cataract is bilateral although often an asymmetric disease [48]. In those cases, bilateral VA is determined by the VA of the better eye. We also had to combine co-morbidities into rather large groups, as new diagnoses during the 11-year follow-up are scarce for many specific diseases. In the longitudinal setting, the right-censoring may have an effect on the results, although this has been tried to minimize by the weighting scheme. Finally, as the study population was predominantly Finnish, the results may not be applicable to other countries and ethnicities, although our use of UK time-trade-off weights for EQ-5D may improve the comparability.

In the future analyses, more large, population-based studies are required to validate the generalization of our results into other settings. Furthermore, additional longitudinal studies with over 10 years of follow-up are needed to ascertain the longitudinal effect of the eye diseases and declining VA on QoL.

In conclusion, our results show that common eye diseases have a declining effect on HRQoL, mental health, and distance VA. However, the decline in HRQoL is not directly affected by the awareness of the eye disease but more likely by the declined VA associated with these diseases. The overall association of these diseases with HRQoL and mental health has decreased between years 2000 and 2011. Furthermore, during the 11-year follow-up newly diagnosed eye diseases showed minor effect on these parameters. This has important clinical implications. As the number of people affected by vision-threatening eye diseases is increasing due to the aging and growth of older population, it is important to prevent the increase of visual impairment caused by these diseases. Our results suggest that the spreading of awareness of the potential hazards of vision-threatening diseases possess very little effect on these parameters compared to the benefits of early diagnosis of these diseases, and therefore should be strengthened to prevent the declining effect of visual impairment on quality of life and increasing healthcare costs.

## Supplementary Information

Below is the link to the electronic supplementary material.

Supplementary file1 (PDF 168 KB)

## Data Availability

Full study protocol, contact details, publications, and the process for collaborating and data requests can be found on the website (thl.fi/health2000).
