# Supplementary material for: Prevalence and 11-year incidence of common eye diseases and their relation to health-related quality of life, mental health, and visual impairment
Source: Qual Life Res. 2021 Mar 23;30(8):2311–27. doi: 10.1007/s11136-021-02817-1 (PMC8298234; doi:10.1007/s11136-021-02817-1)
Supplement: Supplementary file 1 — Supplementary file1 (PDF 168 KB) [file 11136_2021_2817_MOESM1_ESM.pdf]

Prevalence and 11-year incidence of common eye diseases and their relation to health-related quality of life, mental health, and visual impairment

Quality of Life Research

Petri K M Purola, Janika E Nättinen, Matti U I Ojamo, Seppo V P Koskinen, Harri A Rissanen, Päivi R J Sainio, and Hannu M T Uusitalo

Corresponding author:

Petri K M Purola

Department of Ophthalmology, Faculty of Medicine and Health Technology, Arvo building, 33014 Tampere University, Finland

E-mail: petri.purola@tuni.fi

Online Resource 1 Multivariable linear regression analysis examining the changes in EQ-5D and 15D index values, and GHQ-12 total scores between 2000 and 2011

	Change in EQ-5D (n = 1955)		Change in 15D (n = 2158)		Change in GHQ-12 (n = 2302)	
	B coefficients	Beta coefficients	B coefficients	Beta coefficients	B coefficients	Beta coefficients
Constant	0.456 ***		0.384 ***		1.659 ***	
Age at the baseline	–0.0003	–0.020	–0.0005 ***	–0.077 ***	–0.025 ***	–0.081 ***
Male gender	0.001	0.003	–0.004	–0.033	–0.103	–0.017
Incident glaucoma	0.020	0.015	0.001	0.002	0.102	0.004
Incident cataract, unoperated	–0.010	–0.012	–0.014 *	–0.046 *	0.258	0.016
Incident RD	–0.057	–0.037	**–0.020**	–0.036	0.452	0.016
Incident heart disease	–0.034 **	–0.063 **	**–0.017 *** **	–0.083 ***	0.586 ***	0.057 ***
Incident pulmonary disease	–0.035 *	–0.051 *	**–0.020 *** **	–0.078 ***	0.289	0.023
Incident vascular disease	–0.023	–0.032	**–0.017 ** **	–0.062 **	0.528 *	0.038 *
Incident musculoskeletal condition	–0.051 ***	–0.141 ***	–0.009 ***	–0.068 ***	0.345 **	0.051 **
Incident hypertension	–0.035 ***	–0.083 ***	–0.008 *	–0.047 *	0.508 ***	0.063 ***
Incident diabetes	–0.029	–0.038	**–0.021 *** **	–0.072 ***	0.692 **	0.048 **
Incident psychiatric disorder	**–0.091 *** **	–0.121 ***	**–0.040 *** **	–0.139 ***	2.141 ***	0.149 ***
Incident cancer	0.024	0.030	–0.001	–0.004	–0.189	–0.012
QoL index value / total score at the baseline	**–0.491 *** **	–0.444 ***	**–0.381 *** **	–0.383 ***	–0.738 ***	–0.649 ***
R^2	0.222 ***	0.222 ***	0.176 ***	0.176 ***	0.430 ***	0.430 ***
Adjusted R^2	0.216 ***	0.216 ***	0.171 ***	0.171 ***	0.426 ***	0.426 ***

The unstandardized B coefficients show the magnitude of the impact on health-related quality of life and psychological distress, while the standardized Beta coefficients allow the comparison of the explanatory variables with each other. Clinically meaningful B coefficients are bolded (≥ 0.07 for EQ-5D and ≥ 0.015 for 15D). *QoL* quality of life, *RD* retinal degeneration

*Denotes statistical significance with $p < 0.05$

**Denotes statistical significance with $p < 0.01$

***Denotes statistical significance with $p < 0.0001$
